# Molecular Mechanisms of Biotin in Modulating Inflammatory Diseases

**DOI:** 10.3390/nu16152444

**Published:** 2024-07-27

**Authors:** Mika Sakurai-Yageta, Yoichi Suzuki

**Affiliations:** 1 Department of Education and Training, Tohoku Medical Megabank Organization, Tohoku University, Sendai 980-8573, Miyagi, Japan; 2 Advanced Research Center for Innovations in Next-Generation Medicine, Tohoku University, Sendai 980-8573, Miyagi, Japan; 3 Department of Clinical Genetics, Ageo Central General Hospital, Ageo 362-8588, Saitama, Japan

**Keywords:** biotin, holocarboxylase synthase, biotinidase, SMVT, diabetes, proinflammatory cytokine, inflammatory bowel disease

## Abstract

Biotin, also known as vitamin B7 or vitamin H, is a water-soluble B-complex vitamin and serves as an essential co-enzyme for five specific carboxylases. Holocarboxylase synthase (HCS) activates biotin and facilitates its covalent attachment to these enzymes, while biotinidase releases free biotin in the biotin cycle. The transport of biotin, primarily from the intestine, is mediated by the sodium-dependent multi-vitamin transporter (SMVT). Severe biotin deficiency leads to multiple carboxylase deficiency. Moreover, biotin is crucial to glucose and lipid utilization in cellular energy production because it modulates the expression of metabolic enzymes via various signaling pathways and transcription factors. Biotin also modulates the production of proinflammatory cytokines in the immune system through similar molecular mechanisms. These regulatory roles in metabolic and immune homeostasis connect biotin to conditions such as diabetes, dermatologic manifestations, and multiple sclerosis. Furthermore, deficiencies in biotin and SMVT are implicated in inflammatory bowel disease, affecting intestinal inflammation, permeability, and flora. Notably, HCS and probably biotin directly influence gene expression through histone modification. In this review, we summarize the current knowledge on the molecular aspects of biotin and associated molecules in diseases related to both acute inflammatory responses and chronic inflammation, and discuss the potential therapeutic applications of biotin.

## 1. Introduction

Biotin, also known as vitamin B7 or vitamin H, is a water-soluble B-complex vitamin that is essential for the survival of all living organisms as a cofactor for biotin-dependent carboxylases. Mammals, being unable to synthesize biotin, must obtain it from their diet. The biotin cycle plays a crucial role in maintaining the balance between utilizing dietary biotin and recycling endogenous vitamin supplies, thereby ensuring an adequate supply of biotin [1]. Within this cycle, holocarboxylase synthase (HCS; encoded by the *HLCS* gene) and biotinidase (BTD) are pivotal [2,3,4,5]. BTD catalyzes the release of dietary biotin by hydrolysis of biotinylated peptides or biocytin (biotinyl-lysine) during intestinal digestion and the recycling of cellular biotin [6]. HCS converts biotin into its active form, biotinyl-AMP, in an ATP-dependent manner [7]. Subsequently, HCS uses biotinyl-AMP to covalently attach biotin to a specific lysine amino acid within a highly conserved methionine-lysine-methionine sequence in carboxylases. Biotin acts as the co-enzyme for five carboxylases, participating in key reactions in gluconeogenesis, fatty acid synthesis, and amino acid catabolism [1,8,9]. Pyruvate carboxylase (PC) is a pivotal mitochondrial enzyme that converts pyruvate to oxaloacetate, crucial for gluconeogenesis, lipogenesis, and the biosynthesis of neurotransmitters in the brain. Another mitochondrial enzyme, propinyl-CoA carboxylase (PCC), plays a critical role in branched chain amino acids and odd-chain fatty acids catabolism by converting propinyl-CoA to malonyl-CoA. Methylcrotonyl-CoA carboxylase (MCC), another mitochondrial enzyme, is essential for leucine and isovaleric acid catabolism, converting 3-methylcrotonyl-CoA to 3-methylglutaconyl-CoA. Acetyl-CoA carboxylases (ACCs), which include ACC-1 and ACC-2, convert acetyl-CoA to malonyl-CoA, a process that is crucial for fatty acid biosynthesis. ACC-1 predominates in the cytoplasm of liver and kidney, supporting fatty acid synthesis, while ACC-2, which is found on the mitochondrial membrane in muscle cells, regulates mitochondrial fatty acid uptake and oxidation.

Biotin has been shown to not only act as a co-enzyme in metabolism but also to modulate intracellular signaling pathways and the expression of enzymes involved in metabolic processes. Biotin has been implicated in various inflammatory diseases, yet the underlying mechanisms remain to be fully elucidated. This review aims to summarize the molecular pathways modulated by biotin and delve into the uncovered regulatory mechanisms in broadly defined inflammation and inflammatory diseases, including acute immune responses and chronic inflammation, and to discuss the potential therapeutic implications.

## 2. Molecular Mechanisms Involving Biotin

### 2.1. Biotin in Metabolism

Biotin, upon cellular uptake, exerts regulatory control over a multitude of target genes. Significantly, biotin influences the expression of crucial components involved in biotin uptake and metabolism, including sodium-dependent multivitamin transporter (SMVT), also known as solute carrier family 5A6 (SLC5A6), and HCS [10,11]. In contrast, BTD is not involved in these processes. The active form of biotin, biotinyl-AMP, has been reported to play a role in intracellular signal transduction [10,12]. Studies utilizing specific inhibitors and analogues of signaling molecules have demonstrated that biotinyl-AMP activates soluble guanylate cyclase (sGC). This activation subsequently leads to the production of the second messenger cyclic guanosine monophosphate (cGMP) and the activation of downstream protein kinase G (PKG), resulting in the expression of *SMVT* and *HLCS*.

Furthermore, biotin exerts multifaceted effects on glucose and lipid metabolism by modulating the expression of genes that encode pivotal enzymes in biotin-mediated metabolic pathways, including glucokinase [13,14], pyruvate kinase [15], 6-phosphofructokinase [16], and ornithine transcarbamylase [17], as well as various carboxylases (PC, PCC, ACC-1) through the sGC-cGMP-PKG pathway [11,12]. This modulation is also mediated by transcription factors to enhance glycolysis, glycogenesis, and fatty acid degradation via b-oxidation. Biotin supplementation in mice was reported to enhance insulin secretion, increase mRNA levels of glucokinase and insulin, and upregulate key transcription factors, such as forkhead box A2 (Foxa2), pancreatic and duodenal homeobox 1 (PDX-1), and hepatocyte nuclear factor 4α (Hnf4α) [18]. In addition, biotin supplementation was reported to improve β-cell function and proportion while reducing the expression of neural cell adhesion molecule 1 (NCAM-1) [19]. Although NCAM-1 primarily plays a role in maintaining islet architecture, its reduction does not significantly impact glucose tolerance or insulin secretion, indicating that NCAM-1 may have additional functions yet to be uncovered. At pharmacological concentrations, biotin supplementation in mice reduces serum triglyceride concentrations by downregulating lipogenic genes, including ACC and fatty acid synthase in the liver and adipose tissues. The reduction is linked to decreased expression of the transcription factor *sterol regulatory element binding protein 1c (SREBP1c)* [20].

Another transcription factor potentially regulated by biotin is forkhead box O (FOXO1), an important target of insulin action. When insulin levels are low during the fasting state, FOXO1 stimulates the expression of gluconeogenic genes while suppressing genes involved in glycolysis and lipogenesis, including glucokinase and SREBP1c [21]. These multifunctional effects of FOXO1 are exerted through its direct binding to the promoter regions of target genes, as well as its interactions with other transcription factors and co-activators/repressors, depending on insulin levels. Biotin administration was reported to suppress *Foxo1* expression in the liver of type I diabetes rats [22], while no change was observed in *Foxo1* expression in the liver and adipose tissue of biotin-supplemented mice [20]. Although no direct evidence was reported, biotin might inactivate FOXO1 through mechanisms other than transcriptional repression, such as by transportation from the nucleus to the cytosol [23,24].

Furthermore, in 3T3-L1 mouse adipocytes, pharmacological concentrations of biotin were found to decrease fatty acid synthesis and increase fatty acid oxidation and uptake. This was accompanied by the upregulation of mRNA for fatty acid transporters, specifically, *Slc27a1/Fatp1* and *Acsl1* [25]. Additionally, biotin treatment led to an increase in the inactive form of both ACC-1 and ACC-2 and the active form of adenosine monophosphate (AMP)-activated protein kinase (AMPK), which has been shown to be activated by phosphorylation in response to low-energy conditions. Similarly, studies using *Btd*-deficient mice revealed ATP defects, concurrent with the activation of AMPK and inhibition of mammalian target of rapamycin (mTOR), along with augmentation of insulin sensitivity [26]. These findings highlight the significant involvement of post-transcriptional regulatory mechanisms alongside the transcriptional ones.

In terms of amino acid metabolism, biotin-dependent MCC and PCC are involved in the catabolism of leucine and branched-chain amino acids such as isoleucine, threonine, and valine. Biotin deficiency was reported to reduce amino acid levels in mice, particularly sulfur-containing amino acids such as methionine and cysteine, likely due to negative feedback from propionyl-CoA and pyruvate, which are substrates for MCC, PCC, and PC [27]. These findings indicate the intricate role of biotin in regulating amino acid metabolism, with implications for understanding its physiological impact and potential biomarkers of biotin status.

### 2.2. Biotin Transport

The intestine plays a central role in regulating biotin homeostasis, serving as the primary route for biotin acquisition through the release and absorption of dietary biotin and biotin synthesized by intestinal bacteria [1]. Biotin is transported across the cell membrane primarily by the SMVT [28,29], which facilitates the uptake of biotin, pantothenic acid, and lipoic acid. Additional biotin carriers with different kinetic properties and tissue localization have been proposed as well [29,30,31,32,33], although they may make a modest contribution. In peripheral blood mononuclear cells, biotin is absorbed through monocarboxylate transporters, likely due to its chemical similarity with monocarboxylic acids such as lactate and pyruvate [34].

Interestingly, a prioritization of biotin access to the brain during scarcity is observed, similar to the prioritization of the brain during starvation through an increase in insulin-independent glucose transporters [1,11]. Specifically, in experimental biotin deficiency and fibroblasts from a biotin-deficient patient, enzymes related to biotin transport and utilization are downregulated in the liver and kidney during biotin deficiency, whereas in the brain, they remain unaffected.

### 2.3. Biotin and Its Transporter in Cell Structure

As previously described, biotin supplementation induces insulin secretion [35] and affects pancreatic islet architecture, accompanied by a decrease in NCAM-1 protein expression [19]. A study involving normal mice supplemented with biotin for 8 weeks demonstrated notable effects on liver cell morphology without evident markers of liver damage, oxidative stress, or antioxidant enzyme activity [36]. However, another study reported a reduction in oxidative stress after biotin supplementation in the liver of type I diabetes (T1D) mice [37]. These findings suggest that the effects of biotin on cell morphology, cellular architecture, and cellular damage may vary depending on the metabolic status of the cell.

Moreover, biotin was reported to play a crucial role in lipid droplet formation in 3T3-F442A adipocytes, where it required the activation of lipogenic enzymes and the reorganization of the cytoskeleton [38]. In the absence of biotin, no triglyceride accumulation was observed, while the intermediate filament, vimentin, remained in a cytosolic net-like structure. Upon addition of biotin, vimentin redistributed to the periphery of the nascent lipid droplet during early differentiation stages. These findings suggest that biotin may regulate cell structure through changes in the expression and intracellular localization of proteins involved in cell adhesion and the cytoskeleton.

Several studies have highlighted the connection between cell structure and the membrane-bound biotin transporter, SMVT. In a study using *Pals1*-haploinsufficient mice, in which Pals1 is involved in renal cell polarity in the juxta-membrane domain, a lethal phenotype developed, accompanied by heavy proteinuria and renal cyst formation [39]. Transcriptome analysis of these mice revealed the upregulation of target molecules of the TGF-β pathway and the downregulation of SLCs, including SLC5A6/SMVT, although the segment-specific expression and subcellular distribution of SLCs were maintained. Another study identified an association between SMVT and the PDZ-domain-containing protein PDZD11, also known as plasma membrane calcium ATPase-interacting single-PDZ protein (PISP) as well as ATPase-interacting PDZ protein (AIPP1), using yeast two-hybrid screening [40]. PDZD11 was also found to interact with pleckstrin homology domain-containing family A member 7 (PLEKHA7), which plays a role in the stabilization of adherens junctions at juxta-membrane domain [41]. These findings suggest that cell structure may influence biotin uptake by affecting its primary transporter and vice versa.

### 2.4. Biotin and HCS in the Nucleus

In addition to its roles in intracellular signaling and the modulation of transcription factors, HCS and HCS-mediated biotinylation plays a crucial part in nuclear transcriptional regulation. In *Drosophila*, HCS was associated with the heterochromatin domain, colocalizing with the transcriptionally repressive mark, histone H3K9 methylation. HCS indirectly was reported to bind to the core promoter region of *hsp70* gene, sequestering TFIIH subunits and inhibiting RNA Pol II elongation [42]. Subsequent in silico prediction and experiments using yeast and cultured cells revealed the interaction of HCS with euchromatic histone lysine *N*-methyltransferase 1 (EHMT1), leading to reduced H3K9 methylation by HCS knockdown [43]. Moreover, HCS was shown to repress transcription through the histone deacetylases (HDACs) HDAC1, HDAC2, and HDAC7 in HepG2 [44], while HCS overexpression reduced H3K9 acetylation, correlating with transcriptional repression in long terminal repeats and alpha satellite repeats [45]. HCS interacted with HDAC1 and the nuclear receptor co-repressor (N-CoR) within HDAC-containing protein complexes.

These findings support the role of HCS in nuclear transcriptional regulation, but the necessity of biotinylation for transcriptional repression remains unclear. Although one study showed the dispensability of HCS’s biotin ligase activity for HDAC-mediated transcriptional repression [44], another study reported that biotinylation of HDAC1, N-CoR, and EHMT1 correlated with transcriptional repression in an in vitro assay involving ATP and biotin [43,45]. Furthermore, in glioblastoma, HCS-mediated histone biotinylation and acetylation were associated with the modulation of gene expression [46]. The chemical agent sulconazole (SN), chosen for its anti-glioma stem cell properties, disrupted biotin distribution to the carboxylases and histones. Specifically, SN treatment suppressed histone biotinylation and acetylation, reducing the expression of super-enhancer-associated genes critical for glioma stem cells, including *SOX10* and *NEU4*, which were also observed by *HLCS* knockdown. Biotinylation was also observed in the extracellular heat shock protein 72 (HSP72), with this process being dependent on HCS. Culturing HEK293 cells with biotinylated HSP72 resulted in an increase in mRNA expression of *RANTES/CCL5*, indicating a potential regulatory function of this biotinylated protein in the modulation of gene expression [47]. Thus, biotin and HCS play critical roles in gene expression through protein modifications, thereby impacting various cellular processes and potential therapeutic avenues.

## 3. The Roles of Biotin in the Immune System

Biotin deficiency significantly impacts the development and function of lymphocytes in lymphoid tissues. Mice fed a biotin-deficient diet for 20 weeks showed impairments in lymphocyte maturation. Specifically, there was a decrease in the absolute number of splenocytes and the B cell population in the spleen [48] as well as arrested T cell maturation at the double-negative stage [49]. Patients with multiple carboxylase deficiency (MCD) showed defects in T cell and B cell immunity, including a subnormal percentage of T lymphocytes in peripheral blood and no antibody response to pneumococcal polysaccharide immunization [50]. Transgenic *Btd*-deficient mice fed a biotin-deficient diet exhibited cutaneous symptoms and immune dysregulation, including an increase in CD4-positive cells within splenocytes as well as diminished in vitro lymphocyte proliferation [51,52].

Moreover, biotin impacts cytokine expression during various inflammatory stimulations by modulating different intracellular signaling pathways and transcription factors, similar to how it affects the expression of metabolic enzymes. Biotin deficiency was reported to exacerbate nickel-induced allergy in a mouse model, with increased levels of the proinflammatory cytokine IL-1β in splenocytes [53]. Similarly, biotin deficiency augmented proinflammatory cytokine TNF-α production in J774.1 murine macrophage-like cells following stimulation by lipopolysaccharide [54]. Culturing primary human CD4-positive T cells under biotin-deficient conditions resulted in elevated levels of proinflammatory cytokines such as TNF, Th1 cytokine IFN-γ, and Th17 cytokines IL-17, along with active phosphorylated mTOR, a key regulator of cell growth, metabolism, and survival [55]. Additionally, this was accompanied by increased expression of the transcription factors T-bet and RORγt, which upregulate the expression of Th1 and Th17 cytokines, respectively, resulting in a decrease in the proportion of Treg cells and a reduction in the expression of their transcription factor, Foxp3. In contrast, human peripheral blood mononuclear cells (PBMCs) cultured with biotin supplementation for 3 weeks and stimulated with concanavalin A for 21 h exhibited upregulated expression of Th1 cytokine IFN-γ and Th17 cytokine IL-1β [56]. These controversial effects of biotin-deficient and biotin-supplemented conditions on cytokine expression indicate a complex interplay that depends on the types of lymphocytes, stimulation, and biotin status.

Other than T-bet and RORγt, transcription factors regulated by biotin included Sp1/Sp3 and NF-κB, as previously described [57]. Compared with biotin deficiency, biotin supplementation in Jurkat cells, which are immortalized T cell lymphocytes, increased the nuclear abundance and transcriptional activity of Sp1/Sp3, which are GC-box binding proteins that act as transcriptional activators and repressors [58]. Biotin supplementation also elevated IL-2 mRNA levels [59]. Furthermore, biotin supplementation reduced the expression of *sarcoplasmic/endoplasmic reticulum calcium transport ATPase* (*SERCA3*), which is involved in calcium transport, via the Sp1-binding domain [60]. This reduction in *SERCA3* expression was also associated with higher IL-2 and IL-2R protein expression in T cells stimulated by phorbol myristate acetate and ionomycin [61], suggesting a regulatory role of biotin in IL-2 expression through Sp1-mediated transcriptional suppression of *SERCA3*.

Furthermore, biotin-deficient cultured Jurkat cells exhibited increased nuclear abundance and activity of NF-κB upon stimulation, resulting in the expression of genes associated with anti-apoptosis, thereby facilitating cell survival [62]. However, another experiment using J774.1 cells showed no difference in NF-κB activity under biotin-deficient conditions with inflammatory stimulation, despite increased TNF-α production [54]. This discrepancy may arise because the same transcription factor targets genes with varying physiological functions in cells under different exogenous stimulations, necessitating further investigation.

## 4. Biotin in Disorders

### 4.1. Biotin-Dependent Disorders

Biotin-dependent inherited metabolic disorders, resulting from autosomal recessive mutation in the *HLCS* or *BTD* gene, lead to MCD, as summarized in previous reviews [1,9]. HCS deficiency severely reduces the activity of all biotin-dependent carboxylases, impacting various metabolic processes. Symptoms typically emerge early in life and include manifestations such as ketolactic acidosis, organic aciduria, hyperammonemia, along with feeding difficulties, seizures, developmental delay, and metabolic imbalances. Manifestations related to immune inflammation, including alopecia, scaly and erythematous dermatitis, eczema, and candida infections, are also characteristic in MCD [63,64,65]. Pharmacological doses of biotin effectively reverse these symptoms. Most *HLCS* mutations lead to MCD are localized to the carboxyl-terminal, which contains the biotin-ligase domain [3,4,66], impairing HCS function and the activation of the sGC-cGMP-PKG pathway, thereby reducing *HLCS* expression [12].

The *BTD* mutation causes juvenile and late-onset MCD, both of which exhibit symptoms similar to HCS deficiency. Some patients also experience neurological symptoms such as mental retardation and hearing loss. Pharmacological doses of biotin can improve or reverse most symptoms but not the neurological ones. The exact reason for this is unknown; however, in BTD deficiency, the reduced availability of biotin due to BTD mutation may impair *HLCS* expression through the sGC-cGMP-PKG pathway, potentially leading to developmental neurological disorders when both BTD and HCS deficiencies are present [67,68].

Other conditions responsive to biotin treatment include SMVT deficiency and biotin-thiamine-responsive basal ganglia disease (BTBGD). Mutations in the *SMVT* gene, which affect the transport of biotin, pantothenic acid, and lipoic acid, manifest neurodevelopmental delays, feeding problems, and failure to thrive, with significant improvement upon multi-vitamin treatment [69]. BTBGD is caused by a genetic mutation in *SLC19A3* encoding the transporter for vitamin B1 (thiamine) [70]. Biotin deficiency reduced *SLC19A3* gene expression, suggesting that managing biotin intake could be a method for regulating the expression of *SLC19A3* [71].

### 4.2. Biotin in Diabetes

The regulation of glucose and lipid metabolism by biotin has long been investigated in relation to diabetes, using animal and cell models. In these models, biotin deficiency decreased glucose utilization, whereas biotin supplementation stimulated hepatic and pancreatic glucokinase expression and activation [14,72] and reduced phosphoenolpyruvate carboxykinase (PEPCK) and glucose-6-phosphatase for gluconeogenesis [22]. Furthermore, biotin treatment was found to induce insulin secretion [35] and improved tolerance to glucose and insulin resistance in a model of obesity-related type 2 diabetes (T2D) [73] and mice fed with biotin-deficient diet [74]. However, the latter study indicated that altered insulin signaling was not linked to changes in insulin receptor abundance [74]. Several other studies also demonstrated that biotin treatment decreased serum lipid concentrations [20,25].

Clinical trials have also shown the role of biotin in glucose and lipid utilizations. The daily administration of biotin in T1D patients showed a significant decrease in fasting blood glucose (FBG) levels [75]. In T2D patients, daily oral administration of biotin for 1 month with a probiotics drug revealed an inverse correlation between serum biotin levels and FBG levels, although not with serum insulin levels [76]. Several other studies have demonstrated that pharmacological concentrations of biotin can reduce serum triglyceride concentrations in T2D and hyperlipidemia patients [77,78], suggesting a possible role in improving obesity. However, another study showed no significant change in plasma glucose, insulin, or triglycerides after 4 weeks of treatment [79]. The difference among these trials may reflect the heterogeneity of patients as well as variations in administration methods, highlighting the need for further clinical trials considering these points.

Although the relationship between chronic kidney disease, diabetic kidney disease, and biotin has not been extensively studied, research has shown that plasma biotin levels are either normal or higher in patients undergoing chronic hemodialysis [80]. Furthermore, in hemodialysis patients experiencing muscle cramps, plasma biotin and total avidin-binding substances, including bisnorbiotin and biotin sulfoxide, are elevated [81]. Interestingly, these patients showed a limited response to further biotin administration, suggesting that elevated biotin metabolites lacking coenzyme activity may interfere with biotin’s function.

### 4.3. Biotin in Allergic Disorders

In a study of human allergic disorders, patients with atopic dermatitis exhibited significantly lower serum biotin concentrations compared with healthy controls, and percutaneous treatment with biotin-containing ointment reduced peripheral eosinophil numbers [82]. Furthermore, biotin has been implicated in alopecia, an autoimmune disorder associated with mental health as well as allergic conditions such as eczema, hay fever, and asthma [83]. Experimentally, mice fed a biotin-deficient diet showed weight loss after 7 weeks, along with the development of alopecia, and this was prevented by the simultaneous supplementation of biotin in drinking water [84].

In contrast, a positive correlation between serum biotin levels and cedar pollinosis has been observed, contradicting previous findings that indicated a suppressive role of biotin in allergic inflammation [85]. This discrepancy may arise from the analysis of ostensibly healthy individuals in that study rather than extreme cases, or from differences in the measurement of biotin levels in serum as compared with immune cells, indicating the need for further investigation to elucidate the underlying mechanisms and implications.

### 4.4. Biotin in Multiple Sclerosis

Multiple sclerosis (MS) manifests as progressive autoimmune demyelination of axons in the central nerve system [86]. It has been suggested that high-dose biotin might protect against neuronal degeneration by reducing hypoxia via energy production, or by enhancing myelin repair or synthesis through the activation of biotin-dependent carboxylases [87]. In rat oligodendrocyte progenitor cells, high-dose biotin reduced cell death under glucose-derived conditions, enhanced myelin-like ensheathment, and increased ATP production [88]. In *Abcd1* knockout mice, a model of adrenomyeloneuropathy, high-dose biotin restored metabolic balance and rescued axonal degeneration through normalizing phospho-mTOR levels and repressing SREBP1c [89]. Although some clinical studies using high-dose pharmaceutical-grade biotin for MS have shown patient improvement, not all have yielded consistent results, and the use of biotin in MS treatment faced significant challenges after a setback from the European Medicines Agency in 2017, as summarized in [9]. Moreover, a larger trial did not demonstrate significant improvement in MS disability [90]. A recent smaller clinical trial of biotin for chronic demyelinating peripheral neuropathy, which includes chronic inflammatory demyelinating polyradiculoneuropathy, anti-myelin-associated glycoprotein neuropathy, and Charcot–Marie–Tooth 1a or 1b, demonstrated improvements in various sensory and motor parameters, gait abilities, and nerve excitability parameters [91]. A larger randomized controlled trial is warranted to assess the potential benefits of treating demyelinating diseases with high-dose pharmaceutical-grade biotin.

### 4.5. Biotin in Inflammatory Bowel Diseases

Two main types of inflammatory bowel diseases (IBD) are ulcerative colitis and Crohn’s disease, which are characterized by chronic relapses of intestinal inflammation [92]. A decrease in plasma biotin levels has been observed in Crohn’s disease patients, and biotin supplementation enhanced natural killer activity in these individuals [93,94].

Research into the role of biotin in gut inflammation has primarily involved *Smvt*-deficient mice. In intestinal-specific *Smvt* knockout mice, chronic inflammation was observed predominantly in the cecum, resembling features of IBD [95,96]. This pathological state was associated with increased intestinal permeability and alterations in tight junction (TJ) protein expression. Specifically, there was an upregulation of the leaky marker claudin-2, along with a downregulation of the tight marker zonula occludens-1 (ZO-1). *Smvt* deficiency resulted in severe spontaneous intestinal inflammation, growth retardation, developmental delays, and early mortality within the first 6–7 weeks of life. Similarly, dietary-induced biotin deficiency leads to the development of chronic active inflammation in the cecum, characterized by increased intestinal permeability and alterations in the expression levels of TJ proteins [96]. These findings highlight the significant role of SMVT in maintaining normal mucosal integrity, likely through its function in supplying biotin to cells in the gut mucosa.

Additionally, tamoxifen-inducible intestinal-specific *Smvt* knockout mice exhibited spontaneous intestinal inflammation, characterized by elevated proinflammatory cytokines and increased intestinal permeability [97]. Significant induction of calprotectin, a marker of intestinal inflammation and neutrophil infiltration, and the nucleotide-binding domain and leucine-rich repeat pyrin 3 domain (NLRP3) inflammasome were also observed, along with active phosphorylated NF-κB. Furthermore, in a mouse model of dextran sodium sulfate-induced colitis, biotin supplementation positively contributed to the integrity of the intestinal mucosa by inhibiting NF-κB activation [84]. These findings suggest that biotin suppresses IBD by reducing gut inflammation and preserving the integrity of the intestinal mucosal.

The administration of broad-spectrum antibiotics was reported to ameliorate mucosal inflammation by *Smvt*-deficient mice [97], suggesting the significant influence of the gut microbiota in colitis pathogenesis associated with biotin deficiency. In addition to colitis, treating mice with the antibiotic vancomycin resulted in alopecia development, reduced bacterial diversity, and the accumulation of vancomycin-resistant *Lactobacillus murinus* lacking biotin biosynthesis genes [98]. These bacteria consumed residual biotin in the gut and may have contributed to the development of alopecia symptoms in germ-free mice fed a biotin-deficient diet. These symptoms were reversed by biotin supplementation.

Recent findings highlight a direct link between biotin and the gut microbiota in inflammation [99]. Mice rendered biotin-deficient either through a biotin-deficient diet or by *Smvt* conditional knockout displayed intestinal dysbiosis, including the expansion of opportunistic microbes such as *Klebsiella*, *Enterobacter*, and *Helicobacter*, alongside a reduction in mucus-resident microbes such as *Akkermansia*. Predictive metagenomics analysis suggests that certain microbes increase biotin biosynthesis in biotin-deficient conditions. These findings collectively indicate a crucial role of biotin status in intestinal flora, where biotin is taken up and synthesized, highlighting their role in host vitamin homeostasis and inflammation protection.

## 5. Discussion

**Figure 1:**
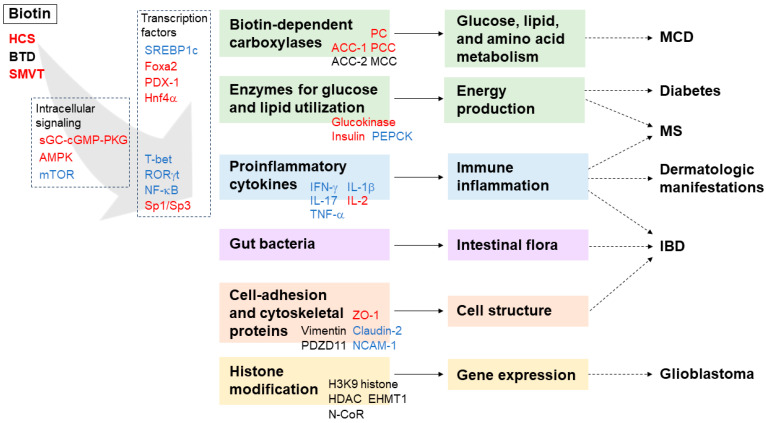
Schematic overview of known molecular pathways that involve biotin. Intracellular signaling molecules and transcription factors affected by biotin are shown in red (activated) or blue (repressed). Categories of downstream targets of biotin with representatives are shown in red and blue for those with positively or negatively regulated expression levels, respectively. The physiological functions of biotin are shown at the ends of the solid arrows. Diseases associated with the disruption of these homeostatic functions are indicated at the ends of the dashed arrows.

This review comprehensively outlines the current understanding of the molecular roles of biotin and its association with diseases characterized by broadly defined inflammation, including acute inflammatory responses and chronic inflammation, as summarized in Figure 1. Beyond serving as a coenzyme for carboxylases, biotin is intricately linked to glucose and lipid utilization in cellular energy production, achieved by modulating the expression of metabolic enzymes such as biotin-dependent carboxylases, glucokinase, and insulin. This modulation is mediated through intracellular signaling cascades, such as sGC-cGMP-PKG, AMPK, and mTOR, as well as transcriptional factors such as Foxa2, PDX-1, Hnf4a, and SREBP1c. Additionally, biotin supplementation is considered to be effective for the improvement of metabolic disorders such as diabetes and hyperlipidemia. Meanwhile, in the immune system, biotin appears to suppress inflammation by inhibiting the production of proinflammatory cytokines under stimulation, partly through common molecular pathways shared with metabolism, such as mTOR signaling. Transcription factors are also involved in these expressions, including T-bet and RORγt in Th1 and Th17 cytokines, Sp1/Sp3 in IL-2, and NF-κB in TNF-α. Reflecting these functions, severe biotin deficiency leads to MCD, which is characterized by neurological defects, possibly due to energy deficits, along with inflammatory manifestations, including impaired lymphocyte development and cutaneous symptoms. The response to high-dose biotin therapy in MS and related demyelinating neuropathies would involve the same mechanisms, including mTOR and SREPB1c, for ATP production and inflammation suppression. Furthermore, SMVT is crucial for the uptake of biotin from the intestinal membrane and is also associated with juxta-membrane proteins involved in cell architecture, suggesting a connection between biotin and cell structure. IBD caused by a deficiency of either biotin or SMVT interacts with immune inflammation and the cellular architecture. Moreover, the effect of biotin deficiency on the gut microbiota may exacerbate IBD. Additionally, HCS and biotin likely play a direct role in gene expression within the nucleus through histone modifications, which might be a fundamental function of biotin and could potentially be associated with its various functions. In summary, the multifaceted functions of biotin are intricately interconnected, including some that remain unknown, and contribute to the orchestration of homeostasis in mammals.

The mechanism underlying biotin’s control of inflammation is expected to become clearer as research extends to encompass other relevant molecular pathways and networks. For instance, the biotin-activated sGC-cGMP pathway, which influences gene expression such as HCS and metabolic enzymes, has been associated with a decrease in blood pressure in spontaneously hypertensive stroke-prone rats, independently of nitric oxide [100]. This pathway demonstrates versatility in generating different phenotypes. Further molecular analyses will provide insights into more precise mechanisms by which biotin regulates metabolism and other phenotypes governed by the common molecular pathways. Another example is the biotin transport pathway. The essential role of the SMVT in facilitating biotin uptake from the intestinal membrane is evident from the observations of biotin in *Smvt*-deficient mice, as described in the previous section, emphasizing the crucial localization of SMVT in the apical domain [101]. Given the distinct separation between the apical and basolateral membranes in intestinal epithelial cells, the export of biotin from the basolateral membrane is vital for its transport throughout the body. However, the identity of the basolateral transporter responsible for biotin export remains unknown. Research utilizing the basolateral membrane of rat enterocytes has provided a clue, indicating that this transporter operates independently of sodium ions, unlike SMVT [102]. The identification of the basolateral biotin transporter is eagerly anticipated, as it will significantly enhance our understanding of biotin transport to blood vessels and other tissues.

As our understanding of biotin’s functions has deepened, its association with certain diseases has become apparent, including the dependency of glioblastoma on biotin distribution [46], as described in Section 2.4. A drug with anti-glioma stem cell properties inhibited glioblastoma metabolism, leading to cholesterol depletion and inhibition of oxidative phosphorylation, which in turn caused an energy crisis. This drug also hindered histone biotinylation and acetylation, which are crucial for the gene expression that is essential for glioblastoma survival. Moreover, increased expression of HLCS is correlated with a poor prognosis in glioblastoma, suggesting that targeting biotin-dependent metabolism and the epigenetic pathway might provide a novel therapeutic approach. However, biotin might be utilized to protect the immune cells surrounding tumor cells. As previously described, biotin supplementation has been associated with reduced expression of SERCA3 in immune cells. Studies have shown that decreasing SERCA3 expression—achieved by administering nifetepimine, a dihydropyrimidone—protects lymphocytes against tumor-induced apoptosis, suggesting its potential as an immuno-restoring agent for cancer patients [103]. Similarly, biotin might act as a tumor-suppressive agent by mitigating immune destruction around tumor cells through the reduction of SERCA3. Comprehensive analyses to identify the expression status of biotin and related molecules in tumors and their microenvironment would further elucidate the potential of biotin in tumor therapy.

Although numerous reports suggest that biotin and HCS contribute to the suppression of inflammation, in cases where inflammation transitions to a chronic state and disrupts cellular or tissue homeostasis, they might instead promote inflammation, as observed in chronic hemodialysis patients [81] and glioma [46]. Given that simultaneously upregulated biotin metabolites lacking coenzyme activity may interfere with biotin’s functions, as observed in the former study, it is essential to carefully assess not only biotin but also biotin metabolites in order to identify the physiological role of biotin and HCS in early and chronic inflammation.

Biotin therapy holds significant promise in conditions such as diabetes and MS, owing to its cost-effectiveness and safety profile. Variations in clinical trial outcomes for MS may be due to patient heterogeneity, including genetic backgrounds, variations in pathology, disease severity and duration, underlying health status, dietary factors, and microbiota responses to biotin. Tailoring biotin therapy to these complexities is crucial, necessitating further research into its pathophysiological roles in these diseases and tailored treatment approaches, with a particular focus on molecular mechanisms. For instance, the identification of three endophenotypes of MS with distinct immune signatures [104] suggests that responses to biotin may vary depending on the immunological signature of the individual patient. Moreover, targeting specific biotin-related pathways presents a promising avenue for investigation. For example, inhibiting ACC1, one of five carboxylases that require biotin as a coenzyme, can suppress Th17 cell formation, thereby promoting Treg development [105]. This process was shown to attenuate Th17 cell-mediated autoimmune disease in mouse models.

Furthermore, the use of not just biotin but also biotin complexes has been explored in autism spectrum disorder. In rats exhibiting autism-like behaviors induced by exposure to propionic acid, treatment with a novel biotin salt, magnesium biotinate, showed dose-dependent improvements in sociability deficits, anxiety-like behaviors, and cognitive impairments [106]. This treatment also resulted in reduced oxidative stress and the suppression of proinflammatory cytokines, although the distinct effects of biotin and magnesium were not discernible. Considering the previously described broad yet moderate functionality of biotin, combining biotin with other modalities warrants consideration aimed at enhancing its therapeutic efficacy.

Notably, biotin’s increasing use in pharmacologic doses for approved and off-label purposes may interfere more frequently with clinical diagnostic tests relying on streptavidin-biotin technology, particularly in hormone measurements and high-throughput analytical platforms [107]. Although pretreating plasma samples with streptavidin microbeads can often correct assay results, a few cases caused by anti-streptavidin antibodies may remain problematic and caution should be exercised.

In conclusion, molecular pathways that involve biotin play significant roles in the molecular regulation of various diseases related to inflammation and contribute to maintaining systemic homeostasis. However, comprehensive studies on the functions of biotin pathways are limited, except for some recent incremental research such as the analyses on biotin in IBD by Said et al., [95,96,97,99], and many reports indicate the involvement of biotin pathways in specific contexts. Utilizing novel techniques such as investigating gene expression and epigenetic modification status at the single-cell level would elucidate the functions of biotin in physiological and disease conditions in various tissues and organs as well as their relevance throughout the body. These approaches would also reveal the effects of biotin deficiency and shed light on the potential therapeutic benefits of biotin supplementation.
